# Flexion Angles of Finger Joints in Two-Finger Tip Pinching Using 3D Bone Models Constructed from X-Ray Computed Tomography (CT) Images

**DOI:** 10.1155/2020/8883866

**Published:** 2020-09-10

**Authors:** Satoshi Shimawaki, Yoshiaki Nakamura, Masataka Nakabayashi, Hideharu Sugimoto

**Affiliations:** ^1^Mechanical Systems Engineering Course, Department of Fundamental Engineering, Utsunomiya University, Tochigi 321-8585, Japan; ^2^Department of Radiology School of Medicine, Jichi Medical University and Hospital, Tochigi 329-0498, Japan

## Abstract

The motion analysis of two-finger tip pinching using the thumb and index finger provides crucial data for designing the motion mechanism of electric prosthetic hands. The purpose of this study is to determine the joints that have high mobility during two-finger tip pinching by measuring the flexion angle of each joint. Ten Japanese men with normal hand were selected. CT images were obtained while the hands adopted the following four postures: a basic posture not pinching a cylinder, and three postures pinching wooden cylinders with different diameters (2, 10, and 30 mm). Three-dimensional bone models of the thumb and index finger were created using the CT images and used to measure the flexion angles of the joints. The flexion angles of the proximal interphalangeal and metacarpophalangeal joints of the index finger significantly decreased as the diameter of the cylinder increased. However, even when the diameter of the cylinder changed, the flexion angle of the distal interphalangeal joint of the index finger, and the flexion and rotation angles of all of the thumb joints did not change. When pinching objects of different sizes with a two-finger tip pinch, the posture of the thumb is fixed, and only the posture of the index finger changes. When designing the two-finger tip pinch motion for an electric prosthetic hand, it is sufficient to drive the joints of the index finger only.

## 1. Introduction

When designing an electric prosthetic hand that accurately simulates human motion with a high degree of freedom, it is necessary to compromise on certain other factors including weight, mechanism simplicity, robustness, and cost [1]. Achieving the most appropriate product overall is therefore a trade-off, and it follows that the factors mentioned can be improved by reducing the degree of freedom while ensuring accurate replication of human movement. One such approach is to use a coupling mechanism that allows two joints to be moved with one motor by finding a special relationship between the flexion angles of the two related joints and connecting these joints with a linkage mechanism. Reducing the number of motors is expected to reduce both the weight and cost. In our previous report [2], we measured the joint angles of each finger when cylinders with different diameters were gripped with a power grip and obtained the coupling ratio between the flexion angles of the distal interphalangeal (DIP) joint and the proximal interphalangeal (PIP) joint of each finger. Another method is to fix a specific joint of the finger at an arbitrary angle. Weight reduction, mechanism simplicity, and robustness can be achieved by reducing the number of joints in the electric prosthesis that need to be driven.

In order to apply joint coupling or joint fixation to an electric prosthetic hand, it is necessary to measure the human motion. Based on interviews with amputees and a survey of activities of daily living (ADL), Cipriani et al. [3] reported that the grip patterns required for electric prostheses were mainly the power grip (35% of ADL), precision grip (30% of ADL), lateral grip with the thumb and index finger (20% of ADL), and extension grip (10% of ADL). We studied power grip in a previous report [2]; therefore, in this study, we focus on the precision grip. Many commercially available prostheses employ a precision grip mechanism. The precision grip is a means of gripping an object using the tips of the thumb and a plurality of fingers. The most basic grasping form of the precision grip is the two-finger tip pinching using the thumb and index finger [4]. This grip is used to pinch small objects or perform delicate and precise tasks with the tips of the thumb and index finger in opposition.

The index finger has DIP, PIP, and metacarpophalangeal (MP) joints distally, and the thumb has interphalangeal (IP) and MP joints distally. Because the DIP and PIP joints of the index finger and the IP joint of the thumb are hinge joints, they have one degree of freedom (flexion-extension) [5, 6]. The MP joints of the index finger and thumb have two degrees of freedom (flexion-extension and adduction-abduction) because they are universal joints. However, because adduction-abduction of the thumb MP joint is known to have a small range of motion, the thumb MP joint is considered to have one degree of freedom (flexion-extension) [6, 7]. In addition, it is known that in the finger tip pinch operation, the adduction-abduction angle of the index finger MP joint does not change when the width of the grasped object is 5.5 cm or less. The index finger MP joint is therefore considered to have one degree of freedom (flexion-extension) [8] and can be regarded as having uniaxial motion during the two-finger tip pinch motion. In addition, the thumb adopts the opposite position to the index finger in the two-finger tip pinch, which is realized by the movement of the trapeziometacarpal (TM) joint of the thumb. The TM joint performs complex movements and typically has 2 degrees of freedom (anterior-posterior translation and flexion-extension) [9, 10].

The purpose of this study was to identify the joints with high mobility during the two-finger tip pinching by measuring the flexion angle of each joint. It was anticipated that by driving only the highly movable joints and fixing the other joints, it would be possible to design a lightweight robust electric prosthetic hand, which has a low degree of freedom but provides biomechanically representative human motion. X-ray computed tomography (CT) of the fingers was therefore performed when cylinders with different diameters were grasped by the two-finger tip pinching using the thumb and index finger, and the flexion angle of each joint was measured using bone models of the thumb and index finger constructed from the CT images.

## 2. Materials and Methods

### 2.1. Subjects

The subjects comprised 10 Japanese men (21–25 years old) who had no hand trauma or disease with a mean height of 169.2 ± 4.2 cm (mean ± standard deviation), a mean weight of 61.3 ± 5.1 kg, and a mean body mass index of 21.4 ± 1.8. The mean hand length, palm length, hand width, index finger length, and thumb length of the subjects were 182.8 ± 2.4 mm, 104.2 ± 2.5 mm, 84.0 ± 2.3 mm, 69.6 ± 3.5 mm, and 62.0 ± 2.8 mm, respectively. This study was approved by the Institutional Review Board for Research on Human Subjects of Utsunomiya University (Approval no.: H18-0011). Before performing the experiment, subjects were given thorough explanations of the purpose and details of the study, and each subject provided written consent.

### 2.2. CT Imaging and Three-Dimensional Bone Model Construction

**Figure 1:**
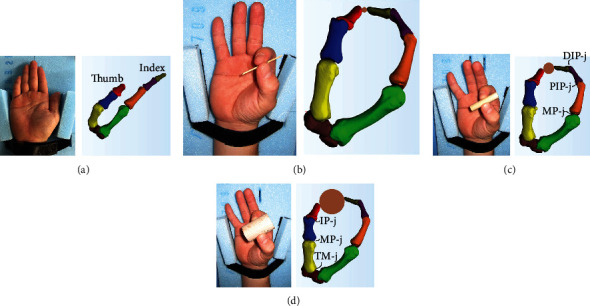
Postures during CT (computed tomography) scanning and in the three-dimensional bone models. (a) basic posture, (b) pinching a 2 mm diameter cylinder, (c) pinching a 10 mm diameter cylinder, and (d) pinching a 30 mm diameter cylinder.

Cross-sectional images from the center of the forearm to the distal portion (spatial resolution: 512 × 512) were obtained with an effective field of view of 150 mm and slice thickness of 0.6 mm using an X-ray CT scanner (SOMATOM Definition AS, Siemens AG, Munich, Germany). The X-ray tube voltage was 120 kV, and the tube current was 150 mA. The rotation time was 0.5 s/rot. CT images were obtained while the hands adopted the following four postures: a basic posture not pinching a cylinder, and three postures pinching wooden cylinders with different diameters (2, 10, and 30 mm) (Figure 1). The cylinder with a diameter of 30 mm weighed 30 g (length: 50 mm). The mode of prehension was a two-finger tip pinch of the side of the cylinder using the index finger and thumb. During CT imaging, the forearm distal portion, forearm central portion, and upper arm distal portion were immobilized with a band and jig so that only the five fingers and palm could move. Subjects were instructed to hold their wrist in a neutral position. Radioulnar flexion was restricted using a holder fixed to the jig, and bending was restricted by ensuring contact between the back of the hand and the jig.

Three-dimensional bone models (the phalanxes of the thumb and index finger, first and second metacarpals, and the trapezium and trapezoid of the carpal) were created using three-dimensional construction software (Mimics, Version.21, Materialise) as shown in Figure 1. The segmentation procedure for creating a three-dimensional bone model was as follows: (i) extract regions with a CT value of 226 HU or more from all images, (ii) remove unnecessary regions visually, and (iii) stack the extracted regions.

### 2.3. Flexion Angles of the Finger Joints

**Figure 2:**
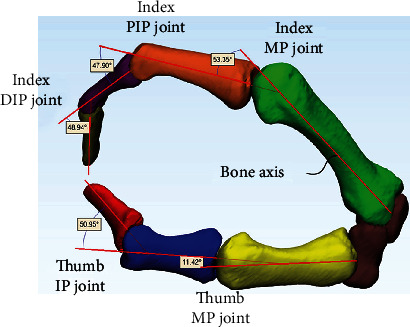
Method for calculating the flexion angles of the thumb and index finger joints.

Bone models in the posture of pinching each cylinder were used to measure the flexion angles of the DIP, PIP, and MP joints of the index finger and the IP and MP joints of the thumb. The method used to calculate the flexion angle of each joint was as follows (Figure 2); the constructed three-dimensional bone models were imported into three-dimensional CAD software (3-matic, Version.13, Materialise), and the bone axis line of each bone model was acquired using a function of the software. The angle formed by the unit vectors of two adjacent lines was defined as the flexion angle.

### 2.4. Rotation Angle and Axis of the Trapeziometacarpal (TM) Joint of the Thumb

When subjects moved from the basic posture to pinch a cylinder, the thumb moved in opposition. The first metacarpal produced a rotational motion with one axis near the TM joint. As the rotation axis was unclear, a finite helical axis representation was used to determine the opposition motion of the thumb. The relative displacement of a moving segment from one position to another can be described in terms of a rotation around and translation along the helical axis. The numerical descriptions used in this representation are explained in the references [11, 12]. The parameters required to specify the first metacarpal movement were the helical axis, which was the rotation axis, the rotation angle around the helical axis, and the extent of parallel translation along the helical axis.

Calculating the parameters was a two-step procedure. In the first step, bone models in two postures—a basic posture and a posture pinching a cylinder—were imported into three-dimensional CAD software (3-matic), and the registration function of the software was used to match the first metacarpals in the two postures. Eight characteristic points were then visually extracted from the matched bone model surface of the first metacarpal. The positions of the characteristic points were made up of three points from each of the proximal and distal portions of the model and two points from the shaft portion. The selected characteristic points were marked at the same position on the surface of the bone models in both postures. The coordinates of the characteristic points differed for each subject. In the second step, the bone models of the trapezium in two postures were matched using the registration function of the software. Using the characteristic point position data (eight points for each posture) marked in the bone models of the first metacarpal in two postures, the helical axis parameter (rotation angle and rotation axis) of the first metacarpal with the transition from the basic posture to the posture pinching a cylinder was calculated.

In order to verify the accuracy and reproducibility of calculating the rotation angle using this method, four sets of different characteristic points were prepared for three subjects, and four rotation angles were obtained using the same method.

### 2.5. Statistical Analysis

One-way repeated measures ANOVA and multiple comparisons using the Bonferroni method (independent variable: diameter) were conducted for the flexion angle of the joints of the index finger and thumb and the rotation angle of the TM joint of the thumb as the dependent variable (SPSS Statistics, Version.22, IBM). Three levels were included in the diameter factor: 2 mm, 10 mm, and 30 mm. The significance level *α* = 0.05.

The accuracy of the TM joint rotation angle calculation was obtained by mean coefficient of variation. Its reproducibility was determined by the intraclass correlation coefficient ICC (1, 1) using the one-way random effect model.

## 3. Results

The average coefficient of variation in the rotation angle of the TM joint was 3.4%. The reproducibility ICC (1, 1) was 0.983, 0.985, and 0.990 for cylinder diameters of 2, 10, and 30 mm, respectively. Therefore, since the ICC (1, 1) was >0.9, the reproducibility of this method can be judged to be excellent. This result shows that high reproducibility is achieved even when the rotation angle is obtained using one characteristic point set.

**Figure 3:**
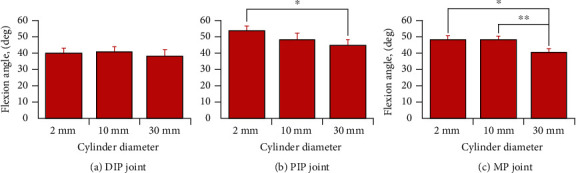
Mean flexion angle of each index finger joint when cylinders of different diameters were pinched. ^∗^*p* < 0.05, ^∗∗^*p* < 0.01. The error bars indicate the standard error.

Figure 3 shows the mean flexion angle at each joint (DIP, PIP, and MP) of the index finger when the three cylinders with different diameters were pinched. The error bar indicates the standard error. There was no significant difference between the flexion angles of the DIP joint when any of the cylinders were pinched (*F*_2_, _18_ = 0.593, n.s.). The mean flexion angles (± standard error) of the DIP joint when pinching 2, 10, and 30 mm diameter cylinders were 39.9° ± 3.3°, 40.7° ± 3.5°, and 38.2° ± 4.1°, respectively. The flexion angle of the PIP joint when the 30 mm diameter cylinder was pinched was significantly smaller than that when the 2 mm diameter cylinder was pinched (*F*_2_, _18_ = 8.419, *p* < 0.001). The mean flexion angles of the PIP joint when pinching 2, 10, and 30 mm diameter cylinders were 54.1° ± 3.0°, 48.5° ± 4.0°, and 44.9° ± 3.6°, respectively. The angle of flexion of the MP joint when the 30 mm diameter cylinder was pinched was significantly smaller than those when the 2 and 10 mm diameter cylinders were pinched (*F*_2_, _18_ = 13.313, *p* < 0.001). The mean flexion angles of the MP joint when pinching 2, 10, and 30 mm diameter cylinders were 48.5° ± 2.5°, 48.4° ± 2.4°, and 40.8° ± 2.5°, respectively. The results showed that the PIP and MP joints of the index finger were moved and adjusted to postures that made it easy to pinch when cylinders of different diameter were grasped. In addition, it was shown that the DIP joint tended to be fixed.

**Figure 4:**
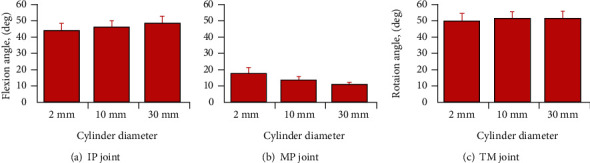
Mean flexion and rotation angle of each thumb joint when cylinders of different diameters were pinched. The error bars indicate the standard error.

**Figure 5:**
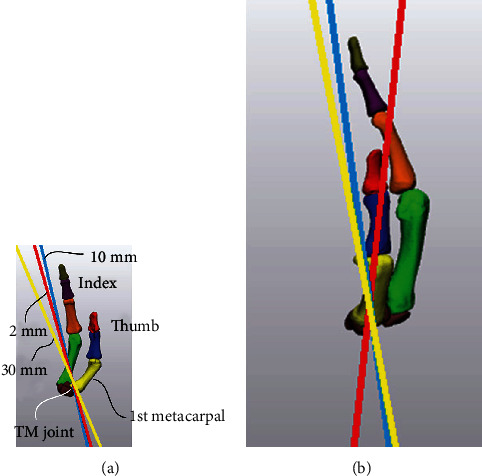
The rotation axes of the 1st metacarpal when pinching cylinders with 2, 10, and 30 mm diameters. (a) Volar view and (b) lateral view.

Figure 4 shows the mean flexion angle or rotation angle at each joint (IP, MP, and TM) of the thumb when the three cylinders with different diameter were pinched. No significant differences were observed in any of the joints (IP: *F*_2,18_ = 1.578, n.s.; MP: *F*_2,18_ = 2.868, n.s.; TM: *F*_2,18_ = 0.198, n.s.). This result shows that the flexion angle and rotation angle of each joint tended to be fixed, even if the diameter of the cylinder changed. The mean flexion angles of the IP and MP joints were 46.8° ± 2.1° and 13.9° ± 1.6°, respectively. The mean rotation angle of the TM joint was 51.2° ± 2.5°.  Figure 5 shows the rotation axis of the TM joint in a typical subject. The rotation axis showed little change, even when the cylinder diameters were different. Figures 4 and 5 show that the posture of the thumb was almost fixed when a cylinder of any diameter was pinched.

It was found that, in case of two-finger tip pinching with the index finger and thumb, in response to the change in the diameter of the pinched cylinder, the postures of the thumb and the DIP joint of the index finger were fixed, and the MP and PIP joints of the index finger were driven.

## 4. Discussion

Several studies have measured the joint angles of the thumb and index finger during the two-finger tip pinching [8, 13, 14]. In these studies, multiple reflective markers were attached to the dorsal surface of the hand, and the flexion angle of each joint was measured using the VICON system. Sakai et al. [14] measured the actions of pinching a 3 mm diameter cylinder with the thumb and index finger. They measured flexion angles at the DIP, PIP, and MP joints of the index finger and the IP and MP joints of the thumb. The flexion angles measured at the IP and MP joints of the thumb did not differ markedly from the results of this study; however, the flexion angle at the DIP joint of the index finger was smaller than in this study, and the flexion angle at the MP joint of the index finger was larger than in this study. One factor contributing to this difference could be that the pinching form measured by them appeared to be the two-finger pulp pinch. Domalain et al. [8] measured the flexion angle at the DIP, PIP, and MP joints of the index finger and the IP joint of the thumb and the abduction and adduction angle at the TM joint of the thumb. The pinched item was not a cylinder, but objects with different widths (3.5–9.5 cm) and masses (0.75–2.25 kg). Two-finger pulp pinching was used. They investigated the relationship between the width of the pinched object and the flexion angle of each joint. The results showed that the flexion angles in all of the index finger joints changed significantly depending on the width of the object; however, those of the thumb did not show significant change. The results for the thumb were consistent with those of the current study. Similar results for the thumb joint have been reported by Yokogawa and Hara [15] and Nakazawa et al. [16]. That is, in the case of two-finger pinching with the index finger and thumb, even if the size of the pinched object changes, the posture of the thumb does not change, and the posture of the index finger corresponds to the change.

The present study suggests that in the case of pinching objects of different sizes with the two-finger tip pinch—without moving both the thumb and index finger—the posture of the thumb is fixed, and only the posture of the index finger changes, which is a human-like movement. Based on this result, it can be concluded that it is sufficient to attach the drive mechanism to the joints of the index finger only when designing the drive mechanism for the two-finger tip pinching in an electric prosthetic hand. This study showed that when cylinders up to 30 mm in diameter are pinched, the DIP joint of the index finger is fixed, and the drive mechanism is only required at the PIP and MP joints. The reason why the two-finger tip pinching operation mainly utilizes the joints of the index finger is not yet clear. Harding et al. [17] assumed that the finger posture was determined to optimize the objective function in terms of finger strength and joint force.

This study was limited because it was only possible to measure the flexion angles of the joints when the cylindrical object was pinched and not during the motion of pinching the object. In contrast, when using the video photography method, the joint angles can also be measured during prehension before a cylinder is pinched [18]. In addition, the experimental design was limited by the following factors. (i) Only two-finger tip pinching was measured. This resulted in some discrepancies when the results were compared with those of other researchers [8, 14]. When a lightweight cylindrical object is pinched, either a finger tip pinch or a finger pulp pinch can be used. In the present study, the two-finger tip pinch was selected, but it is necessary to evaluate the two-finger pulp pinch in future studies. (ii) The cylinder diameter used in this study was limited to 30 mm. Other researchers [8, 13] used cylinders with larger diameters. However, when the diameter of the cylinder increases, the mass of the cylinder increases, and it is thought to be difficult to pinch greater masses with the two-finger tip pinch.

## 5. Conclusions

The flexion angles of the proximal interphalangeal and metacarpophalangeal joints of the index finger significantly decreased as the diameter of the cylinder increased. However, even when the diameter of the cylinder changed, the flexion angle of the distal interphalangeal joint of the index finger, and the flexion and rotation angles of all of the thumb joints did not change. When pinching objects of different sizes with the two-finger tip pinch, the posture of the thumb is fixed, and only the posture of the index finger changes. When designing the two-finger tip pinch motion for an electric prosthetic hand, it is sufficient to drive the joints of the index finger only.

## Data Availability

The data that support the findings of this study are available on request from the corresponding author, Satoshi Shimawaki.
